# Assessment of the incidence and etiology of nosocomial diarrhea in a medical ward in Iraq

**DOI:** 10.25122/jml-2021-0275

**Published:** 2022-01

**Authors:** Ammar Jabbar Hamad, Aseel Jassim Albdairi, Samer Nema Yassen Alkemawy, Safaa Ali Khudair, Noor Rafea Abdulhadi

**Affiliations:** 1. Department of Medicine, College of Medicine, University of Kufa, Al-Najaf, Iraq; 2. Department of Physiology, College of Medicine, University of Kufa, Al-Najaf, Iraq; 3. Department of Medicine, Al Sadar Medical City, Al-Najaf, Iraq

**Keywords:** diarrhea, nosocomial infections, gastrointestinal parasites, medical ward

## Abstract

This study aimed to detect the incidence, etiology, risk factors, and severity of nosocomial diarrhea among adult inpatients in a medical ward in Iraq. The study was conducted among patients admitted to the medical ward from June 1, 2019, to January 31, 2020, in AL-Sader medical city. The surveillance for nosocomial diarrhea was performed by monitoring every patient in the ward 3 times/week. 1050 patients were admitted to the medical ward in AL-Sader medical city. Of these, 52 patients (mean age 58±12.91 years, range 32 to 80) developed new-onset diarrhea during hospitalization. There was a significant relationship between the severity of diarrhea and age, residence, antibiotic use, including number and duration of antibiotics, immunosuppressive agents (steroids/chemotherapy), duration of hospital stay, level of consciousness, and enema use. Nosocomial diarrhea is a significant clinical problem that complicates about 5% of all admission in the medical ward. Various microorganisms account for nosocomial diarrhea, including *E. histolytica*, *G. lamblia*, and *Candida*. Several risk factors associated with the severe form of nosocomial diarrhea include old age, antibiotic use, immunosuppressive use, and length of hospital stay.

## Introduction

Diarrhea is one of the most common gastrointestinal problems encountered in the outpatient, emergency departments, and hospitals. There are many infectious, dietary/drug, gastrointestinal, extra-intestinal, and surgical causes. Understanding the physiology and pathophysiology of nutrient digestion and intestinal absorption can guide the diagnostic approach [1]. Diarrhea may be described as increased stool fluidity or increased stool frequency that causes urgency or abdominal discomfort. Diarrhea is defined as stool volume of more than 200g per day over 24–72 hours [2]. Acute diarrhea persists for less than 2 weeks. Diarrhea has multiple osmotic, secretory, and exudative mechanisms [3–4], and it can be caused by more than one of these mechanisms. Therefore, it is clinically useful to classify diarrhea as watery, malabsorptive (fatty), and inflammatory. Diarrhea represents an important mortality cause worldwide, especially for the elderly and children (below five years) in developing countries [5–6]. Ingestion of some poorly absorbed solute (*e.g.*, Mg+2) or unabsorbed (*e.g.*, lactulose) may cause diarrhea. The osmotic force of these solutes pulls water, sodium, and chloride into the lumen of the intestine so that a considerable percent of stool osmolality comes from ingestion of non-absorbed or poorly absorbed solute [7–8]. Hospital-acquired diarrhea may be considered a significant hospitalization risk that occurs in 2–32% of admitted patients in general medicine wards [9–10]. Noninfectious causes of diarrhea, including medications and underlying illness, should be considered by clinicians in most cases of nosocomial diarrhea [11–12]. The frequency of infectious causes such as *Norovirus*, some strains of *Clostridium perfringens*, *Staphylococcus aureus*, and Bacteroides fragilis are still undefined, and tests are limited [12–13]. The risk factors for diarrhea include inadequate hospitalization facilities such as isolation units, bed space, visitors, source of food (outside from restaurant etc). Also, inadequate management of waste, contaminated equipment, and transmission of infection from health workers may be causative factors [14–15].

Acute diarrhea: infectious diarrhea represents about 80% of acute diarrhea cases, while the remaining cases of acute diarrhea are due to medications or other causes [16]. Food-borne and water-borne infectious diarrhea are primarily due to *Salmonella*, *Campylobacter jejuni*, *E. coli*, and *Shigella* [17–18]. Food poisoning diarrhea is most commonly caused by *Staphylococcus aureus*, *Bacillus cereus*, *Clostridium perfringens*, and *Clostridium botulinum* [19]. Food poisoning is caused by toxins in food due to contamination with microorganisms. Antibiotic-associated diarrhea occur in about 20% of hospitalized patients using broad-spectrum antibiotics. About 30% are due to Clostridium difficile, which can cause severe diarrhea [20–21].

Nosocomial diarrhea is most commonly caused by antibiotics and medications associated with diarrhea, *C. difficile* infection, tube feeding problems, or underlying illness. Magnesium-containing laxatives, magnesium-containing antacids, and lacunose may cause osmotic diarrheas [22–23]. Bisacodyl laxatives may cause secretory diarrhea. Liquid formulations of medications cause diarrhea (elixir diarrhea) because of the high content of sorbitol or other non-absorbable sugars (*e.g.*, mannitol) used to sweeten the elixir [24–25]. Immune suppressed patients are also susceptible to nosocomial viral infections (*Rotavirus*, *Norovirus*, *Adenovirus*, and *Coxsackievirus*) [26].

Radiotherapy and chemotherapy-related diarrhea: abdominal or whole-body radiation causes watery bowel movement, chemotherapy using some drugs, *e.g.*, amsacrine, azacitidine, cytarabine, dactinomycin, daunorubicin, may cause mild to moderate diarrhea. Immune checkpoint inhibitors cause diarrhea in up to 40% of patients. Angiotensin-converting enzyme inhibitors may cause diarrhea due to visceral angioedema. Olmesartan causes diarrhea because of sprue-like enteropathy. Cholestyramine, colestipol, and colesevelam result in diarrhea, especially in patients with ileal resection because it binds bile salts [26].

### Classification of diarrhea according to the degree of dehydration

• No dehydration (loss of <3% of body weight);
• Mild degree of dehydration, loss of 3–5% of whole-body weight manifested by dry oral mucosa & thirst;
• Moderate degree of dehydration, loss of >5–9% of body weight manifested by increased thirst sensation with dry oral mucosa and sunken eyes associated with decreased urine output and hypotension with prolonged capillary refilling and dry skin;
• Severe dehydration, *i.e.*, loss of ≥9% of body whole weight with moderate dehydration with hypovolemic shock [27].

### Classification of diarrhea by CTCAE v5.0 (Common Terminology Criteria for Adverse Events v5.0)

• Grade 1: <4 bowel movement in 24 hrs from baseline; mild increase in ostomy output as compared to baseline;
• Grade 2: 4–6 bowel movements in 24 hrs from baseline; moderate increase in ostomy output as compared to baseline;
• Grade 3: ≥7 bowel movements in 24 hrs from baseline; hospitalization indicated; severe increase in ostomy output compared to baseline;
• Grade 4: Life-frightening consequences; insistent interference indicated;
• Grade 5: Death [28].

This study aims to detect the incidence, etiology, risk factors, and severity of nosocomial diarrhea among adult inpatients in a medical ward in Iraq.

## Material and Methods

The medical ward of AL-Sader medical city has a capacity of 70 beds, with nearly 1575 admissions per year. The study was conducted among patients admitted to the medical ward of AL-Sader medical city from June 1, 2019, to January 31, 2020. Surveillance of nosocomial diarrhea was done by visiting all patients in the ward three times a week. The patients were asked about the number of bowel movements per day, consistency, mucous or blood in the stool, and recent alteration in their bowel habits. During the eight months of the study, every patient was assessed to screen the risks of acquiring diarrhea and followed until discharge. Nosocomial diarrhea was diagnosed when a patient with no diarrhea during the previous 14 days before hospitalization had at least 3 watery bowel movements or at least 4 semi-liquid stools in the last 24 hrs for 2 or more days after 3 days of hospital admission.

Exclusion criteria: patients with gastrointestinal diseases, Crohn’s disease or ulcerative colitis, patients who complain of Malena (bloody diarrhea), Celiac disease, or lactose intolerance, and patients on medications that cause diarrhea. After admission to the medical ward, all patients were screened using a questionnaire covering the type of medical illness, drug therapy, duration of admission, history of previous admissions, and diarrhea symptoms. All patients with diarrhea had routine stool examination, stool culture, and other follow-up investigations (e.g., complete blood picture, electrolytes, renal function etc), urine output, hydration level, physical examination, and vital signs. In addition, three swabs were taken from three sites: the patient table, the door handle of the medical ward, and the door handle of a toilet in the medical ward.

### Collection of samples

When stool samples are poorly collected, these will be of little or no value for exact diagnosis, as amoebic trophozoites start to collapse within 1–2 hours of passage. If stool specimens are stored for numerous hours or overnight, especially in a hot climate, the cysts will be damaged. The fecal samples were processed as follows: a portion of fresh stool was mixed with methylene blue and iodine stains to detect parasites, neutrophils, yeasts, and the pseudohyphae.

*Candida* was the leading cause of diarrhea if no other microorganism was present and pseudohyphae were present in a fresh smear and/or a fungal culture originated from the fecal sample. Patients with many yeast forms in the fresh stool smear (215 per high-power field) were recognized as a suspect category. The diarrhea was associated with drugs if no microorganism was recognized and if it happened within7 days of taking laxatives, hypertonic solutions, or enteral feeding. Bacterial microorganism were grouped as usual: *Shigella*, *Salmonella*, *Aeromonas*, enterotoxigenic *E. coli*, and *C. difficile*) or as unusual: *Enterobacteria*, *Klebsiella*, *Morganella*, *Citrobacter*, or *Pseudomonas*).

### Statistical Analysis

SPSS (Statistical package for social sciences) version 26.0 was used to conduct data analysis. Categorical variables were assumed as number and percentage, and continuous variables were assumed as mean±standard deviation. Comparison of study groups was performed using the chi-square test for categorical data and Student’s t-test for continuous data. P-value <0.05 was regarded as a statistically significant value.

## Results

From June 1, 2019, through January 31, 2020, 1050 patients were admitted to the medical ward in AL-Sader medical city. Of these 1050 patients, 52 patients (mean age 58±12.91 years, range 32 to 80) complained of diarrhea during their hospital stay. 24 patients (mean age 48.50±9.99) developed mild diarrhea (no need for intravenous rehydration), and 28 patients (mean age 66.21±8.92) developed moderate-severe diarrhea (with intravenous rehydration). There were 29 (55.7%) male patients and 23 (44.3%) female patients. Out of 52 patients, 29 (55.7%) were from rural areas and 23 (44.3%) from urban areas. There was a significant relationship between the severity of diarrhea and age (p-value<0.001) and residence (p-value 0.009). However, there was no significant relationship between the severity of diarrhea and gender (p-value 0.14), as shown in Table 1.

**Table 1.** The relation of age, gender, and residence with the severity of diarrhea.

**Variables**		**Severity of diarrhea**						**P-value**
		**Moderate-Severe**		**Mild**		**Total**		
		**NO.**	**%**	**NO.**	**%**	**NO.**	**%**	
**Age (Year)**	**>60**	23	44.23	3	5.77	26	50	<0.001
	**41–60**	4	7.69	16	30.77	20	38.46	
	**30–40**	1	1.92	5	9.62	6	11.54	
	**Mean±SD**	66.21±8.92		48.5±9.99		52	100%	
**Gender**	Male	17	32.69	12	23.08	29	55.77	0.14
	**Female**	11	21.15	12	23.08	23	44.23	
**Residence**	Rural	17	32.69	6	11.54	29	44.23	0.009
	**Urban**	11	21.15	18	34.62	23	55.77	

Moreover, there was a significant relationship between antibiotic use and the severity of diarrhea, including the number of antibiotics administered. 27 patients received two antibiotics, and 25 patients received one antibiotic (p-value <0.001). Moreover, there was a statistically significant (p-value 0.012) effect on the duration of antibiotics used (27 patients ≥7 days, 25 patients ≤7days), as shown in Table 2. Regarding immunosuppressive agents (steroids/chemotherapy), 30 patients received immunosuppressive agents (7 patients with cancer: 3 lung cancer, 3 bladder cancer, one leukemia). 22 patients did not receive immunosuppressive agents, and there was a statistically significant effect on diarrhea (p-value <0.001), as shown in Table 2. The analysis of other risk factors, including duration of hospital stay, level of consciousness, and enema use, also revealed a significant effect on the severity of diarrhea, as shown in Table 2.

**Table 2.** The relation of risk factors with severity of diarrhea.

**Variables**		**Severity of diarrhea**						**P-value**
		**Moderate-Severe**		**Mild**		**Total**		
		**NO.**	**%**	**NO.**	**%**	**NO.**	**%**	
Number of antibiotics	**One antibiotic**	14	26.92	11	21.15	25	48.07	<0.001
	**Two antibiotics**	14	26.92	13	25	27	51.92	
Duration of antibiotics	**<7days**	9	17.31	16	30.77	25	48.08	0.012
	**≥7 days**	19	36.54	8	15.38	27	51.92	
**Immune suppression**	**No**	8	15.38	10	19.23	18	34.61	<0.001
	**Yes**	20	38.46	14	26.92	34	65.38	
**Duration of admission**	**<7days**	9	17.31	16	30.77	25	48.08	0.012
	**≥7 days**	19	36.54	8	15.38	27	51.92	
**Enema**	**Yes**	16	30.77	6	11.54	22	42.3	0.019
	**No**	12	23.08	18	34.62	30	57.7	
**Level of consciousness**	**Alert**	13	25	23	44.23	36	69.23	<0.001
	**Confused**	15	28.85	1	1.92	16	30.77	

Screening for medical problems in our patients revealed 21 (40.3%) patients with pneumonia, 17 (32.7%) patients with a recent cerebrovascular accident, and 14 (27%) patients with urinary tract infection. There was no significant difference in the severity of diarrhea among these medical problems (p-value 0.45), as shown in Table 3.

**Table 3.** Relation between medical problems and the severity of diarrhea.

**Type of medical problem**	**Severity of diarrhea**						**P-value**
	**Moderate-Severe**		**Mild**		**Total**		
	**NO.**	**%**	**NO.**	**%**	**NO.**	**%**	
**Pneumonia**	9	17.31	12	23.08	21	40.39	0.45
**(C.V.A)**	16	30.77	1	1.92	17	32.69	
**U.T.I**	3	5.77	11	21.15	14	26.92	

The results of general stool examination(G.S.E) and fecal culture showed 15 samples with *Candida*, eight samples with *E. histolytica*, three samples with *G. lamblia*, and 26 samples with no specific pathogen. There was no significant difference in the severity of diarrhea and isolated pathogens (p-value 0.30), as shown in Table 4.

**Table 4.** The relation between isolated pathogens with the severity of diarrhea.

**Isolated pathogens**	**Severity of diarrhea**						**P-value**
	**Moderate-Severe**		**Mild**		**Total**		
	**NO.**	**%**	**NO.**	**%**	**NO.**	**%**	
*Candida*	5	9.62	10	19.23	15	28.85	0.3
*E. histolytica*	7	13.46	1	1.92	8	15.38	
*G. lamblia*	2	3.85	1	1.92	3	5.77	
*None*	14	26.92	12	23.08	26	50	

In this study, there were 12 swabs from 3 sites (patient’s table, door handle of the ward where the patient developed diarrhea, and door handle of the toilet in the ward). These swabs were collected every two months during the study period. All swabs showed no growth of any specific pathogen.

## Discussion

Nosocomial diarrhea frequently occurs among patients in the medical ward. This type of diarrhea can become a serious problem as the patients are exposed to various factors that may decrease their immunity and aggravate diarrhea [29]. Patients who acquire nosocomial diarrhea have a higher mortality rate than other patients with a higher risk of transmission to other patients [30–31]. Nosocomial diarrhea is associated with appreciable morbidity and mortality in developing countries. This study documents the occurrence, magnitude, and etiological agents of infectious nosocomial diarrhea. Our study found that 52 out of 1050 patients developed hospital-acquired diarrhea compared to the study of Bhuiyan *et al.*, who accounts for 26 patients out of 1,000 [9,32]. Moreover, there was a significant difference in the severity of diarrhea and age, older people being more likely to develop nosocomial diarrhea, which agrees with another study that found old age as an essential risk factor [33]. Another study [34] reported an incidence of nosocomial diarrhea in 3 to 28 adults for every 100 admissions, while in elderly patients over 70 years, it was 17 to 31 for every 100 admissions. Another study identified a relative risk of 6.6, 11.8, and 14.3 for the age groups 41 to 60, 61 to 75, and over 75 years, respectively [34–35]. Furthermore, another study [36] concluded that the extremes of age were more vulnerable to nosocomial diarrhea [37].

There was a significant difference between the severity of diarrhea and residence in our study, with rural areas being more vulnerable to nosocomial diarrhea. This finding corresponds to another study [38], and this may be explained by dietary habits and hygiene. There was no significant difference in the severity of diarrhea and gender, consistent with other results that found no differences in severity of diarrhea between males and females [36]. Several risk factors related to the severity of diarrhea included the number of antibiotics, duration of antibiotics, administration of immunosuppressive agents (steroids/chemotherapy), and length of hospital stay. Chemotherapy or steroids can be risk factors for infective nosocomial diarrhea by depressing cellular immunity against amebic and fungal infection [11]. Our results agree with a study [33] that revealed that the duration of hospitalization is (more than 7 days) related with the development of nosocomial diarrhea.

The number of antibiotics used also affects the development of diarrhea; this can be explained by the effect of antibiotics on the concentration of anaerobic bacteria in the intestine and subsequent reduction in carbohydrate metabolism, resulting in osmotic diarrhea. The risk of nosocomial diarrhea increases with each day of hospitalization after more than 3 days due to an increased risk of exposure to pathogens that may cause a new type of diarrhea [5, 9]. Patients with longer periods of hospitalization with intensive treatments like intensive care unit admission, transplantation, and chemotherapy develop diarrhea at a rate of 15% to 80% [12, 22]. There was a statistically significant relationship between the level of consciousness and the enema used with the severity of diarrhea, which was prevalent among many confused patients. This is consistent with the result of a study [30], which found that mechanical wash away of healthy gastrointestinal flora by enema permit amoebas reactivation and causes diarrhea. Our results are similar to studies [35] that found enemas causes nosocomial diarrhea (iatrogenic cause). We found no significant difference in the severity of diarrhea and the type of medical problems. However, another study [35] showed that pneumopathy, especially community-acquired pneumonia, was most common among patients with diarrhea [33]. There was no significant difference in the severity of diarrhea and isolated pathogens among our patients. These results are comparable with another study that found the most frequently isolated agents were yeasts and *E. histolytica* while bacterial pathogens played a minor role [30]. Another study by Sandokji *et al.* found that enteropathogenic bacteria or protozoa, *e.g.*, *G. lamblia* and *E.* *histolytica*, were the most detected organisms [36]. Some studies revealed that most frequently, the cause of nosocomial diarrhea was a bacterial infection and the use of antibiotics. However, parasite infection was not detected, so in most cases, the cause is unknown [39]. Negative stool culture in our study may be due to viral etiology or *C. difficile* infection, which needs a particular method of stool culture or toxin assay.

## Conclusion

Nosocomial diarrhea is a significant clinical problem that complicates about 5% of all admission to the medical ward. Various microorganisms account for nosocomial diarrhea, including *E. histolytica*, *G. lamblia*, and *Candida*. Several risk factors are associated with the severe form of nosocomial diarrhea, including old age, the number of antibiotics used, immunosuppressive use, and length of hospital stay.

## Acknowledgments

### Conflict of interest

The authors declare no conflict of interest.

### Ethical approval

This study was approved by the Ethics Committee of the College of Medicine, University of Kufa (KTCM-09)

### Consent

Written informed consent was obtained from the participants in the study.

### Authorship

AJH contributed to collecting data and manuscript conceptualization. AJA contributed to writing, data analysis, and manuscript submission. SNYA is the corresponding author and contributed to revision. SAK contributed to data collection. NRA contributed to data collection and data analysis.
